# Contour Fitting of Fused Filaments Cross-Section Images by Lemniscates of Booth: Application to Viscous Sintering Kinetics Modeling

**DOI:** 10.3390/polym13223965

**Published:** 2021-11-16

**Authors:** Laurent Chaunier, Anne-Laure Réguerre, Eric Leroy

**Affiliations:** 1 INRAE, UR BIA, 44316 Nantes, France; anne-laure.reguerre@inrae.fr; 2 Université de Nantes, Oniris, CNRS, GEPEA, UMR 6144, 44600 Saint Nazaire, France; eric.leroy@univ-nantes.fr

**Keywords:** biopolymer, fusion-bonding, morphological image processing, parametric equations, viscous sintering

## Abstract

A method for image analysis was implemented to determine the edge pixels of two biopolymer-based thermoplastic filaments during their hot melt isothermal sintering at 120 °C. Successive inverted ellipses are adjusted to the contour of the sintered filaments and lead to the identification of the parameters of the corresponding lemniscates of Booth. The different steps of the morphological image analysis are detailed, from 8-bit coded acquired images (1 frame/s), to the final fitting of the optimized mathematical functions describing the evolution of the filaments envelope. The complete sequence is composed of an initial pure viscous sintering step during the first minute, followed by viscoelastic swelling combined with melt spreading for a longer time, and then the stabilization of the sintered filaments shape for over 2 min at high temperatures. Using a master curve obtained from Hopper’s abacus, the characteristic viscous sintering time is assessed at *t_vs_* = 78 s, confirming the one previously found based on the measurement of the bonding neck length alone. Then, the full description of the evolution of the thermoplastic filaments envelope is assessable by image analysis during sintering trials as a result of its digital modeling as successive lemniscates of Booth, reflecting geometry changes in the molten state.

## 1. Introduction

This study deals with the modeling of the viscous sintering of thermoplastic filaments based on a 3D printable biopolymer processed in the molten state. In fused filament deposition-based additive manufacturing processes, the local cross-section between successively deposed filament layers may be considered as a representative element for the description of the mesostructure, affecting the porosity and mechanical properties of the printed parts [1,2,3]. However, there is currently a lack of equations for the description of such cross-section geometry, and lemniscate curves may be good candidates. Indeed, such algebraic curves have been successfully used by Hopper for the modeling of ceramic sintering kinetics in the case of cylindrical filaments maintaining a constant area when described as a side view [4]. The author gave an exact analytic solution of the plane-flow case where capillarity drives the evolution of the shape of the two cylinders’ profile as a function of the sintering time. Starting from this assumption, we propose using the viscous sintering of thermoplastic filaments as a model case to demonstrate the ability of the lemniscates of Booth to fit the contour of the local cross-section and to drastically enhance previous approaches only based on bonding neck length.

**Figure 1:**
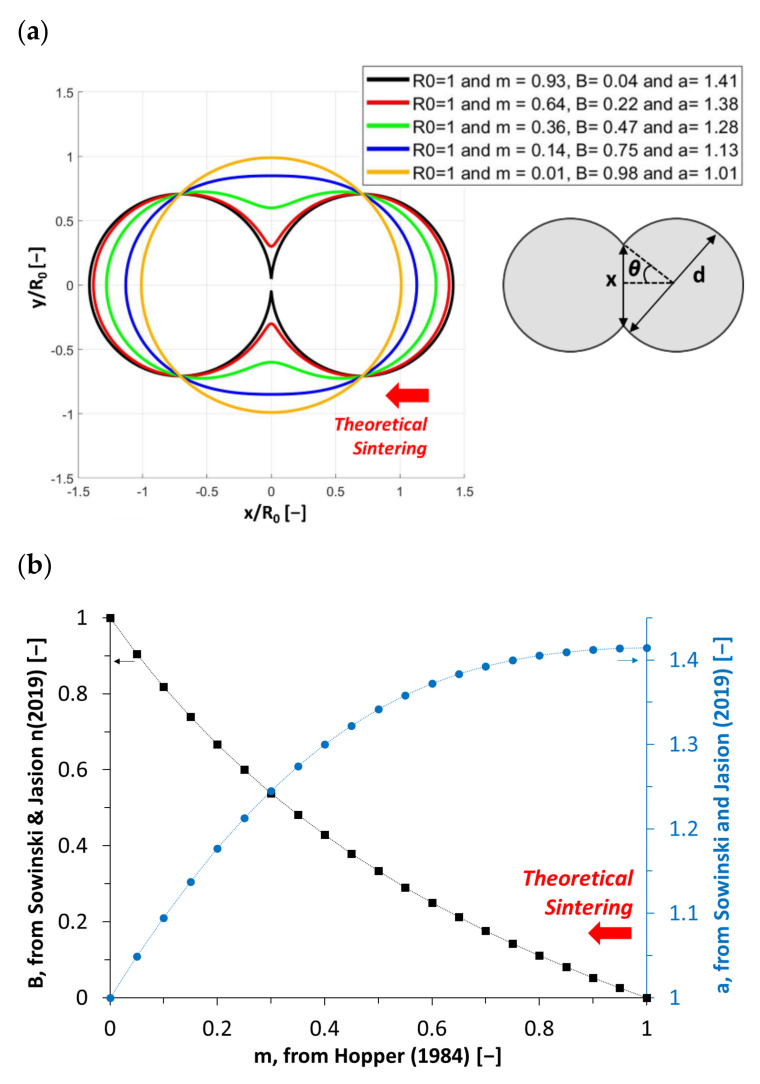
Schematic representation of the theoretical evolution of the contour of two round thermoplastic parts during their viscous sintering as successive inverted ellipses: Lemniscates of Booth presenting a constant area and plotted in a Cartesian coordinate system, with parameter m = 0.93, 0.64, 0.36, 0.14 and 0.01 (Equation (4), with R_0_ = 1), the corresponding parameters in a polar coordinate plane: B = 0.04 (a = 1.41), 0.22 (a = 1.38), 0.47 (a = 1.28), 0.75 (a = 1.13), 0.98 (m = 1.01) -Equation (5)- and illustration of the length of the bonding neck, x, between the two parts of diameter, d, with bonding angle θ (**a**). Theoretical evolution of parameters B and a -Equation (5)-, with m (in the case of R_0_ = 1; from Hopper (1984) [4]) (**b**).

The characterization and modeling of viscous sintering kinetics are experiencing a renewed interest due to the development of additive manufacturing technologies based on thermoplastic materials in the form of filaments or powders [5,6,7]. For this purpose, image acquisition systems are set up to follow the coalescence of such divided solid materials in instrumented ovens, or by thermo-microscopy [8,9,10,11]. This approach relies on evaluating the length of the bonding neck, *x* (m), between two materials as round parts of diameter *d* [m], observed from the side in the case of filaments, or from above in the case of powder particles, and determining their bonding angle, *θ* (rad) (Figure 1a):

(1) $$\theta =\sin^{-1}\left(\frac{x}{d}\right),$$

The growth rate of the bonding angle can be experimentally assessed during viscous sintering trials. It is then possible to determine the characteristic viscous sintering time, *t_vs_* [s], by using the tabulated values from the pioneering work of Hopper [4,12]:

(2) $${\left(\frac{d\theta }{d\left(\frac{t}{t_{vs}}\right)}\right)}_{Hopper}=t_{vs}\times {\left(\frac{d\theta }{dt}\right)}_{Experimental},$$

The term *t_vs_* (s) is linked to the material properties and is defined as follows:

(3) $$t_{vs}=\left(\frac{R_{i}\times µ}{\Gamma }\right),$$

where *R_i_* is the initial radius (m) of the thermoplastic material submitted to sintering trials; μ is the viscosity of the molten material (Pa·s); and Γ is its surface tension (N/m).

The determination of *t_vs_* makes it possible to define the processing window of the considered material, especially to target its additive manufacturing in the molten state. It was recently applied to the deposition of molten filaments based on a thermoplastic biopolymer, zein, plasticized by 20% glycerol leading to edible and resorbable parts, not accessible with synthetic polymers [12]. *t_vs_* was found to drastically decrease, from values above 3500 s when reaching the molten state at 80 °C, to *t_vs_* < 100 s at temperatures above 120 °C (i.e., t_vs_zein20%glycerol_120°C_ = 80 s). This order of magnitude is similar to the one of a standard amorphous polymer, acrylonitrile butadiene styrene (ABS), used in additive manufacturing at 240 °C, its own printing temperature [12,13]. In this case, the viscous sintering time, *t_vs_*, was determined by the application of charts established on the evolution of the bonding neck length between two thermoplastic round parts, i.e., two extruded cylindrical filaments observed from the side (R_i_, [m]) and the relationship to the parametric equations of typical lemniscates curves. These curves represent a series of successive inverted ellipses that evolve during sintering. They correspond to the gradual change in the contour profile of molten round parts [4], as later demonstrated in the literature [14,15]. *t_vs_* was found to be on the same order of magnitude as the one determined by the modified Frenkel model, taking the variations in particle diameter during the coalescence step into account [8,16,17]. However, to take the entire geometry of the observed system into account, beyond the simple evaluation of the length of the melting front between two thermoplastic filaments brought to high temperatures, this approach must be supplemented by the analysis of their contour as a whole. Furthermore, the geometry characterization that is conventionally used is limited to the initial phase of the sintering sequence because of the apparent swelling of the filaments observed from the side, by an edge effect due to the viscoelastic swelling of melts [18] and their spreading due to gravity over a longer time. To improve this approach, we can link the coordinates of all the points of the edge of the filaments to a theoretical lemniscate of Booth adjusted to the closest of these points. It is expressed in Cartesian coordinates, according to Hopper’s approach presented in a vertical layout in his work [4], and adapted in the present work for horizontal juxtaposed filaments:

(4) $$\left\{\begin{array}{c} x\left(\alpha \right)=R_{0}\times \left[\left(1-m^{2}\right)\times {\left(1+m^{2}\right)}^{-\frac{1}{2}}\times {\left(1+2m\times \cos2\alpha +m^{2}\right)}^{-1}\right]\times \left(1+m\right)\times \sin\alpha \\ y\left(\alpha \right)=R_{0}\times \left[\left(1-m^{2}\right)\times {\left(1+m^{2}\right)}^{-\frac{1}{2}}\times {\left(1+2m\times \cos2\alpha +m^{2}\right)}^{-1}\right]\times \left(1-m\right)\times \cos\alpha \end{array}\right.,$$

where α ∈ [0, 2π); R_0_ is the final radius of the inverted ellipse resulting from the coalescence of the two initial circles of radius R_i_ (with R_0_ = √2 × R_i_, set as constant in order to maintain a constant total section area); and parameter m ∈ [0, 1).

Such geometry is also described in a polar coordinate system to give more insight into its properties, as recently proposed by Sowinski and Jasion [19]:

(5) $$r_{p}\left(\varphi \right)=a\times \sqrt{\cos^{2}\varphi +B^{2}\times \sin^{2}\varphi },$$

where the parameter a is the size coefficient, and B is the shape parameter (-). 𝜑 [rad] is defined as α-π/2 from Hopper’s work in a horizontal layout, with the bonding neck placed at x = 0, and where all of the inverted ellipses have a common point of interception at 2^−1/2^ × R_0_ (±1, ±1) (Figure 1a). B ∈ [0, 1], corresponding at the edge of this range to Booth lemniscates describing a circle in the case where B = 1, or to two connected circles with a tangent point centered on the origin point (0,0) for B → 0, or m → 1, in the case where m = 0 in Hopper’s equation (Equation (4); Figure 1b).

The objective of the present study was to set up and implement a novel method for the monitoring of the viscous sintering of cylindrical filaments used in additive manufacturing. It is based on advanced image analysis and modeling of the evolving contour of the filaments’ cross-section during the sintering process. The modeling involves a set of constitutive parametric equations of the successive inverse ellipses that match the contour of the filaments.

Compared to previous studies considering the sole measurement and modeling of the evolution of the length of the bonding neck between the filaments [8,10,12], this new approach not only allows estimating the characteristic time for viscous sintering, but also gives an efficient simulation of the shape of the filaments cross-section. Therefore, it could be used to model the local two-layer geometry in printed parts.

For this purpose, this paper describes: (i) the successive steps that allow the determination of the coordinates of the contour edge points of two juxtaposed backlighted filaments placed in an instrumented oven to monitor their hot melt sintering; (ii) the adjustment of the lemniscate corresponding to their contour on each acquired image using the Sowinski and Jasion equation (Equation (4)) in polar coordinates; and (iii) the fine checking of the characteristic viscous sintering time, *t_vs_*, of the thermoplastic biopolymer, the plasticized zein in this case, used as a model at typical efficient sintering conditions (i.e., T = 120 °C).

## 2. Materials and Methods

### 2.1. Plasticized Biopolymer and Filament Extrusion

The commercial zein powder (Ref. Z3625) and glycerol, used as a plasticizer, were purchased from Sigma-Aldrich (Saint-Quentin-Fallavier, France). They were mixed in a household kneader to obtain the formulation based on zein with 20w% glycerol added. This powder blend was extruded with a twin-screw microcompounder at 130 °C (Haake Minilab, Thermo Scientific GmbH; Karlsruhe, Germany), as previously described [12]. The residence time at 130 °C was typically about 60 s before the extrusion of the plasticized zein through a cylindrical die (Ø_filament_ ≈ 2 mm). Differential scanning calorimetry (DSC) experiments showed that the material presents a glass transition with an onset temperature T_g_ = 42 °C. This is associated to a main mechanical relaxation occurring at about 45–48 °C, as assessed by dynamic mechanical analysis (DMA) at the peak of the loss modulus E″. Above 75–80 °C, the measurement of the moduli is no longer possible in DMA because of material flow. The filaments were cut as cylinders with a constant length, L_filament_ = 5 mm, for subsequent fusion-bonding characterization.

### 2.2. Sintering Trials

An instrumented oven was specifically designed for the sintering of two horizontally adjacent extruded filaments, as detailed in previous work, in perfect alignment with the imaging axis. The front of the cavity is a transparent glass plate, while the back is equipped with three LEDs for the backlighting of the two thermoplastic filaments. A CMOS camera allows the acquisition of contrasted images coded on 8 bits, i.e., 256 grey levels, of the cylinder’s cross-section (1 frame·s^−1^) with an average resolution about 200 pixel/mm.

To monitor the temperature of the filaments (T_filament_, [°C]), a thin thermocouple (type K, Ø_thermocouple_ ≈ 0.4 mm) was placed inside one of them, previously drilled to its half-length to insert the thermocouple tip. Each experimental sintering trial was carried out at T_set_ = 120 °C in order to create an efficient sintering effect while not expanding the material (i.e., bubbles appearing above 135 °C to 140 °C, as described in previous work). A second thermocouple (type K) was located in the medium part of the oven to monitor the environmental temperature (T_oven_, [°C]).

### 2.3. Morphological Image Analysis

**Figure 2:**
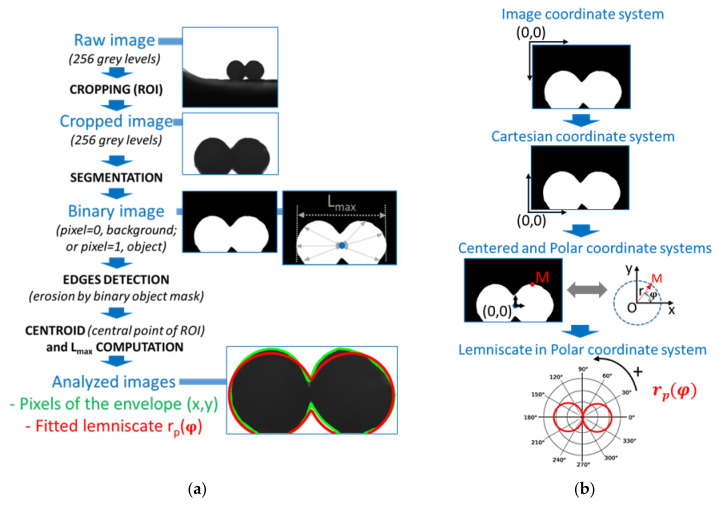
Image morphological analysis to assess the coalescing filaments contour on each acquired image (**—**) and its fitted lemniscate of Booth (**—**) (**a**). Schematic representation of the successive changes from the segmented -binary- image in a standard image coordinate system to a Cartesian centered one and, finally, to a polar coordinate system (with an example of coordinate conversion taken for one point M), as used in the approach of Sowinski and Jasion [19] (**b**).

**Figure 3:**
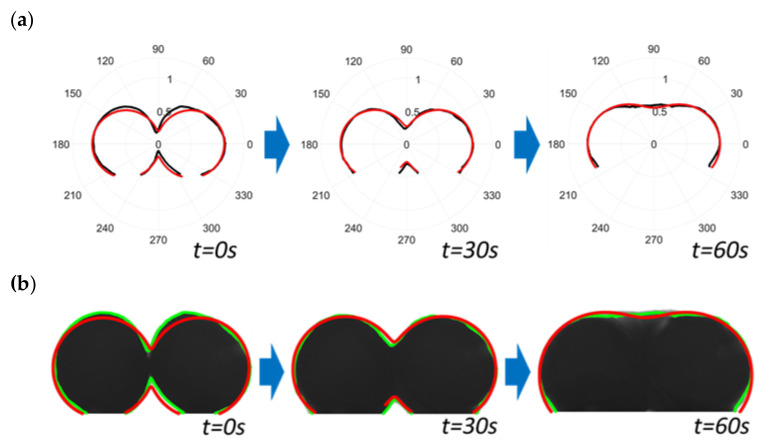
Plot of the successive envelopes of the filaments detected as edge pixels by image analysis (**—**) and the corresponding fitted lemniscates by Matlab^®^ solver (least squares method) (**—**) in a polar coordinate system, as in the approach of Sowinski and Jasion [19] (**a**). Images acquired during the sintering of two thermoplastic filaments at 120 °C (8-bit coded, i.e., 256 grey levels) and superimposition of their edge (**—**) and the successive fitted lemniscates of Booth (**—**) (**b**) (for corresponding video files, please see the Electronic Supplementary Materials: ESM_2 and ESM_3, respectively).

Image analysis is applied on the 8-bit coded images (i.e., 256 grey levels) acquired during the sintering of the two juxtaposed filaments placed at 120 °C (Figure 2a):

- Step 1: The image of the region of interest (ROI) is cropped in order to focus on the two initial filaments and to follow their progressive sintering, while maintaining the initial coding of the image format as 256 grey levels;
- Step 2: A segmentation is carried out by image thresholding in order to obtain binary images with one white object corresponding to the two filaments (i.e., with pixels = 1) on a black background corresponding to the backlighting (i.e., pixels = 0). The constant lighting conditions allow using a single threshold visually determined to segment the whole images sequence with ImageJ software (free software; National Institutes of Health, Bethesda, MD, USA). Based on each binary image, the morphological image analysis (MIA) is applied to determine each point of the contour of the sintered filaments and to fit the adjusted lemniscate to their contour, according to the following steps:
- Step 3: The identification of the contour pixels by an erosion step on the digital mask corresponding to the filament shape with a 3 × 3 pixels^2^ structuring element, excluding the oven base;
- Step 4: The determination of the centroid point, i.e., the equivalent of the center of gravity of the white object of the segmented image;
- Step 5: The computation of the maximum horizontal length, L_max_ [pixel], from one side to the opposite side of the white object on the binary image;
- Step 6: The fitting of a lemniscate of Booth, adapted to the contour of the filaments on each acquired image, implemented on Matlab^®^ software (The MathWorks Inc., Natick, MA, USA). To carry out this last step, a conversion from the initial image coordinate system to a polar centered one is required (Figure 2b and Figure 3a). Each image is vertically centered on the ordinate at which the horizontal length of the white object is maximum (i.e., where L_max_ is determined) and, horizontally, at the abscissa of the centroid, which is equivalent to a central point for the two coalescing filaments.

From the (x, y) centered coordinates of each filaments edge pixel (e.g., point M in the scheme in Figure 2b), the conversion to a polar coordinate system is possible as follows:

(6) $$\left\{\begin{array}{c} x=r\times \cos\varphi \\ y=r\times \sin\varphi \\ r=\sqrt{x^{2}+y^{2}} \end{array}\right.,$$

It is then possible to estimate the parameters of the Lemniscate of Booth equation fitting the contour of the filaments. The least square method of the Matlab^®^ solver was used to optimize the value of the shape parameter B of the polar equation r_p_(𝜑) of the lemniscate, as proposed by Sowinski and Jasion (Equation (5)), while the evolution of the white object’s size during sintering trials is taken into consideration by the size coefficient, a = L_max_/2. To do so, the parameter B is determined by minimizing the distance between the contour’s pixels and the fitted lemniscate using the *fminsearch* function of Matlab^®^ software (please see the script detailed in the Electronic Supplementary Materials section, as ESM_1). Then, the two parameters of the fitted lemniscate, a and B, are assessed and the mathematical function of the fitted lemniscate can be plotted. Its constitutive pixels, as well as the ones of the filaments’ contour, can be superimposed on each corresponding acquired image, after conversion of their coordinates into the image coordinate system and plotting. This leads finally to each analyzed image (Figure 2a and Figure 3b).

## 3. Results

### 3.1. Successive Lemniscates of Booth to Fit Sintering Sequences

The use of the least square method to optimize the equation of lemniscates of Booth in a polar coordinate system makes it possible to identify the shape parameter, B, of the equation r_p_(𝜑) of Sowinski and Jasion (Equation (5)). Curves correctly match the contour of the filaments during their isothermal sintering, even if changes in shape modify their aspect: First, during their initial pure viscous sintering step, up to 50 to 60 s at 120 °C, and then when submitted to viscoelastic swelling and spreading for a longer time (Figure 3a; please see the animation in the Electronic Supplementary Materials section, as ESM_2).

When superimposed on the acquired images in their associated coordinate system, the lemniscates of Booth correspond to the edge pixels of the thermoplastic filaments during the complete sequence (Figure 3b; please see the animation in the Electronic Supplementary Materials section, as ESM_3).

### 3.2. Determination of the Parameters of the Lemniscate Equations

**Figure 4:**
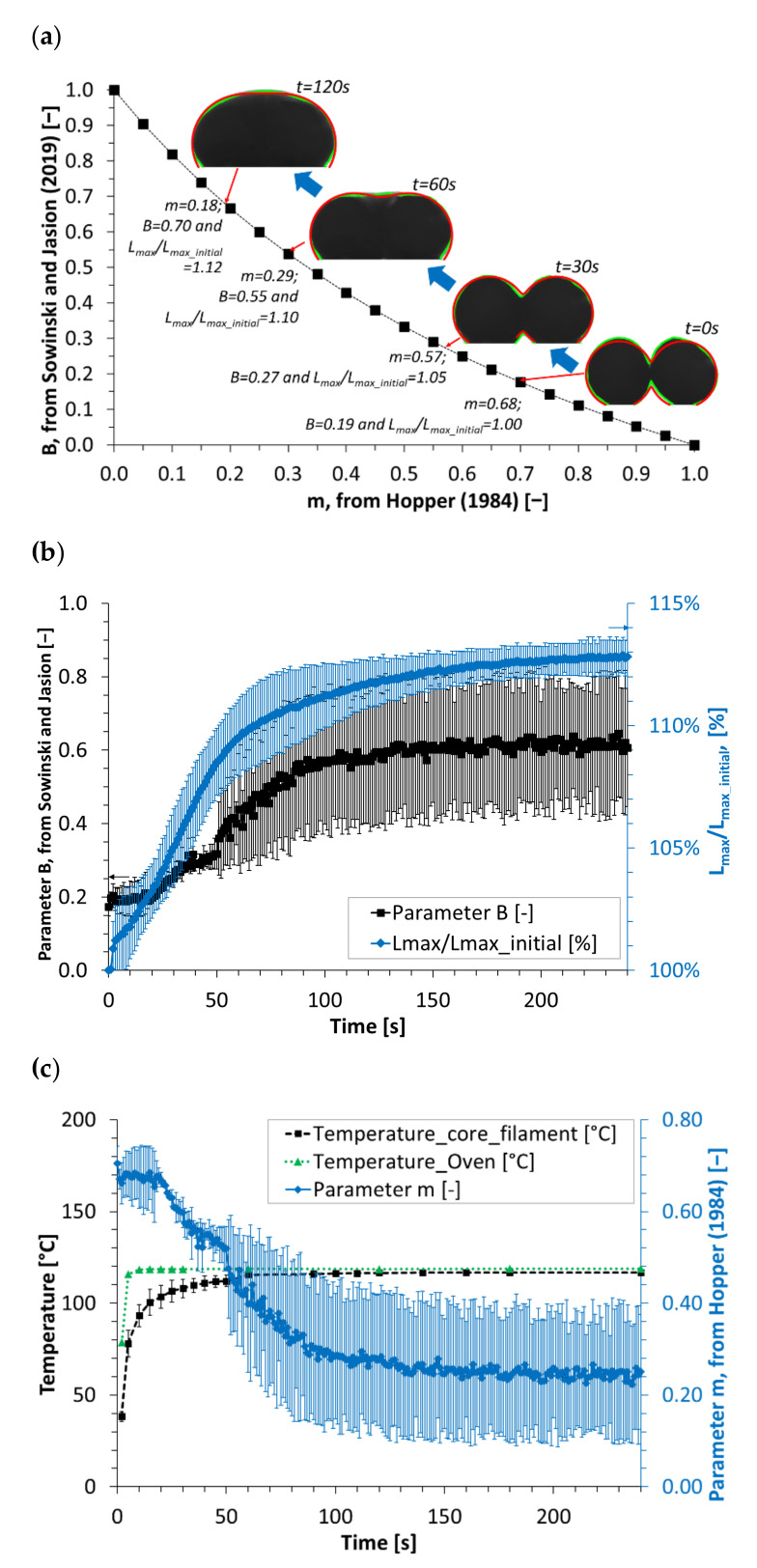
Theoretical evolution of the parameter B (from Sowinski and Jasion [19]) with parameter m (from Hopper [4]). Presentation of the acquired images of the thermoplastic filaments during a sintering trial at 120 °C with the superimposition of their edge pixels (**—**), the successive fitted lemniscates (**—**) and their corresponding values of parameter m (Hopper [4]), B and L_max_ (normalized here as the ratio: L_max_/L_max_initial_, and used to compute the parameter a (Sowinski and Jasion [19]), assessed by image analysis (**a**). Evolution of the parameter B and L_max_ (normalized as L_max_/L_max_initial_), assessed by image analysis during thermoplastic filament sintering at 120 °C (**b**). Evolutions: (i) of temperatures measured in the oven environment; (ii) inside an extruded filament for T_set_ = 120 °C; and (iii) of the values of the parameter m (Hopper [4]), assessed from parameter B (Sowinski and Jasion [19]) (**c**).

Based on the determination of the shape parameter, B, which increases from 0.19 to 0.7 during a 2-min sintering sequence at 120 °C (Figure 4a; Data available as an accessible dataset cited as reference [20]), it is possible to assess the parameter m of the equation from Hopper’s work (Equation (4)), as follows:

(7) $$B=\frac{1-m}{1+m},$$

The monitoring of the coalescence follows the evolution of the shape of the filaments. The values of m decrease from 0.68 to 0.57 (after 30 s), 0.29 (after 1 min) and 0.18 (after 2 min). At the end of the sintering sequence presented here, the increase in the size coefficient is 112% of the initial L_max_ determined on the first image (namely “L_max_initial_” in Figure 4) because of the viscoelastic swelling of the melt and its gravity-driven spreading.

This ratio is stable after about 90 s, as is the shape parameter, typical of the disappearing concavity between the two thermoplastic filaments during their hot melt sintering (Figure 4b). It was checked at the end of each trial carried out at high temperature that the molten sintered parts were not bonded to the metallic surface of the oven when removed.

As detailed in the experimental section, the oven is regulated at T_set_ = 120 °C for these sintering trials. It typically takes 20 s for the environmental temperature, T_oven_ to return to the regulated T_set_ value once the sintering sequence begins, after closing the glass window of the oven (Figure 4c). It takes more time for the filament core to reach the surrounding temperature, but since the sintering primarily takes place at the filament outline, it is assumed that the peripheral material reaches a steady state after about 20 s, when reaching the molten state [12]. This corresponds to the starting point of the increase of the B parameter from Sowinski and Jasion (Figure 4b), or the symmetrical decrease of the deduced m parameter from Hopper’s work (Figure 4c).

### 3.3. Assessment of the Material’s Characteristic Viscous Sintering Time

**Figure 5:**
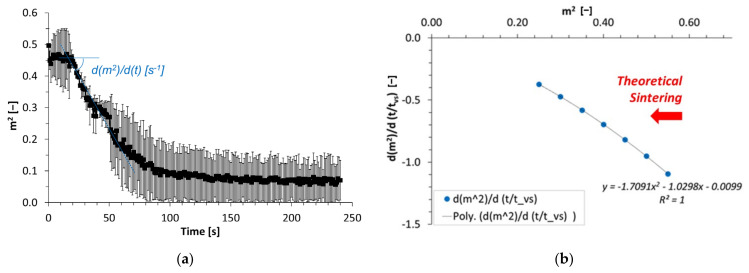
Evolution of the parameter m^2^ during the coalescence of thermoplastic filaments at 120 °C (**a**). Theoretical evolution of the decrease rate of m^2^ in relation to the characteristic viscous sintering time, *t_vs_*, and plotted vs. m^2^ during theoretical sintering (master curve derived from Hopper’s work; Hopper [4]) (**b**).

To determine the characteristic viscous sintering time, *t_vs_*, the use of Hopper’s abacus is based on the evolution of m^2^ (from m of Equation (4)) [4]. It decreases in a symmetric way, like the one of parameter B, and allows the determination of the slope in the pure initial sintering step, from about 20 s to about 1 min at 120 °C (Figure 5a). This experimental slope, (*dm*^2^/*dt*)_*experimental*_, is assessed at −6.0 × 10^−3^ ± 2.4 × 10^−3^ s^−1^. Furthermore, the use of Hopper’s abacus [4] makes it possible to draw a theoretical master curve (Figure 5b) and its polynomial fitting as: (*dm*^2^/*d*(*t*/*t_vs_*))_theoretical_ = *f*(*m*^2^).

For typical values of m^2^ that decrease from 0.4 to 0.2 in the linear sintering domain from 20 to 60 s, this makes it possible to determine the theoretical coefficient (*dm*^2^/*d*(*t*/*t_vs_*))_theoretical_ at −0.4727, extrapolated for m^2^ = 0.3.

This value is linked to the experimental slope by the characteristic viscous sintering time, *t_vs_*, as follows:

(8) $${\left(\frac{dm^{2}}{d\left(\frac{t}{t_{vs}}\right)}\right)}_{theoretical}=t_{vs}\times {\left(\frac{dm^{2}}{dt}\right)}_{experimental},$$

Such result leads to the determination of the characteristic sintering time: *t_vs_* = −0.4727/−0.0060 = 78.8 ± 31.8 s. This value of *t_vs_* may be compared to the one previously assessed at about 80 s by the determination of the bonding neck length between sintered filaments and its associated growth rate [12]. They are similar and this result confirms the efficiency of the novel image analysis method leading to simulate the geometry of thermoplastic parts during their sintering. It also confirms the processing window previously defined for this biopolymer-based thermoplastic material, i.e., the plasticized zein, with the complete assessment of the sintering sequence, now possible, as the progressive decrease of the residual concavity, followed by swelling and spreading for a longer time. The evolution of the estimated parameters required to use Hopper’s model are similar to those that allowed the determination of the bonding neck length alone, but in the present work, it corresponds to the exact monitoring of the filament geometry, progressively transformed into a homogeneous round melt obtained at high temperatures after the complete sintering of the filaments.

## 4. Discussion

In this work, the ability of lemniscates of Booth to describe the contour of the cross-section of a pair of thermoplastic 3D printing filaments has been demonstrated in the model case of viscous sintering. The efficient analysis of images acquired during the viscous isothermal sintering of thermoplastic filaments allows the integral monitoring of the evolution of their overall edge shape. Indeed, such a side view shape follows their pure initial coalescence, up to about 60 s, followed by viscoelastic swelling and spreading up to about 90 s with a radial straining of 112%. The determination of the parametric equations of successive lemniscates of Booth that fit their evolving contour leads to the computation of the characteristic viscous sintering time of the considered material, checked at *t_vs_* ≈ 80 s, as previously found with a standard approach based on the determination of the bonding neck length between sintered parts. These results validate the enhanced images analysis approach proposed in the present work and its use for the assessment of sintering properties of a thermoplastic polymer in the molten state. In future works, the equations of lemniscates of Booth may be used to simulate the cross section’s shape of juxtaposed filaments, as the local geometry of basic patterns constituting digital twins of 3D printed objects.

## Data Availability

Data are available as an accessible dataset cited in the manuscript as reference [20] (12 October 2020) https://data.mendeley.com/datasets/gyd29mpt6b/1.

## Supplementary Materials

The following are available online at https://www.mdpi.com/article/10.3390/polym13223965/s1, Script-Code ESM_1: Script (Matlab^®^) to optimize the value of the shape parameter B of the polar equation of the fitted lemniscate. Video ESM_2: Sintering sequence at 120 °C and corresponding image analysis (1 frame/s acquired over 120 s, accelerated 10 times and saved as a movie file). Plot of the successive shape of the filaments detected as edge pixels by image analysis (**—**) and the corresponding fitted lemniscates of Booth (**—**) in a polar coordinate system. Video ESM_3: Sintering sequence carried out at 120 °C and its corresponding image analysis (1 frame/s acquired over 120 s, accelerated 10 times and saved as a movie file). Images acquired during the sintering of two thermoplastic filaments at 120 °C (8-bit coded, i.e., 256 grey levels) and superimposition of their edge (**—**) and the successive fitted lemniscates of Booth (**—**).
