# Supplementary material for: Contour Fitting of Fused Filaments Cross-Section Images by Lemniscates of Booth: Application to Viscous Sintering Kinetics Modeling
Source: Polymers (Basel). 2021 Nov 16;13(22):3965. doi: 10.3390/polym13223965 (PMC8622888; doi:10.3390/polym13223965)
Supplement: Supplementary file 1 [file polymers-13-03965-s001.zip › ESM_1.pdf]

Online Resource 1. Script (Matlab®) to optimize the value of the shape parameter B of the polar equation of the fitted lemniscate, as proposed by Sowinski and Jasion [20], based on values issued from image analysis: a=L_{max}/2, the size coefficient; and polar coordinates determined from the (x, y) centered coordinates of each filaments edge pixel:

$$\begin{cases} x = r \times \cos\varphi \\ y = r \times \sin\varphi \\ r = \sqrt{x^2 + y^2} \end{cases}$$

, as reference to Eq. 6 in the manuscript.

```
% Matlab® : fminsearch finds minimum of unconstrained multivariable function
% using derivative-free method
B=fminsearch(modelFunctSowinski,0.01);
function [sse, rAdj] = modelFunctSowinski(B)
            % rAdj=a*sqrt(cos²(phi)+B²*sin²(phi))
           rAdj=a*sqrt(power(cos(phi),2)+power((B*sin(phi)),2));
           % error vector
           Error_Vector = rAdj - r;
           sse = Error_Vector(:)'*Error_Vector(:);
end
```
